# A Lecture on Placenta Prævia

**Published:** 1910-04-16

**Authors:** G. E. Herman

**Affiliations:** Consulting Obstetric Physician, London Hospital.


### Hospital Clinics.

### A LECTURE ON PLACENTA PRÆVIA.

By G. E. HERMAN, M.D., F.R.C.P., Consulting Obstetric Physician, London Hospital.

(Delivered at the Medical Graduates' College and Polyclinic, Chenies Street, W.C., on Thursday, March 24, 1910).

Gentlemen,—I speak to you to-day on the subject of placenta prævia. The first thing to which I ask your attention is the physiology of placenta prævia; how the labour naturally goes on. At a period in the pregnancy, the exact date of which we know not—the late Dr. Matthews Duncan thought it was in the last fortnight, but I am sure it is often earlier than that; I have known it occur by the seventh month—the internal os begins to dilate; the cervical canal comes to form part of the general uterine cavity; and the chorion advances through the os internum. In a normal pregnancy the bands of adhesion between the chorion and decidua are very slender, and there is no difficulty about the chorion advancing through the os internum. In a few cases the chorion is attached to the decidua by bands of unusual firmness, and this is an occasional cause of delay in the first stage of labour. By separating the membranes all round the internal os one not only accelerates labour but may sometimes induce it.

The foregoing applies to a natural pregnancy. In pregnancy with placenta prævia you have the placenta attached low down, so that instead of a chorion loosely attached to the decidua you find a placenta which is firmly attached to the uterus. The placenta cannot get into the cervical canal without vessels being broken across where it is attached to the uterus. In placenta prævia the seventh month of pregnancy is about the usual time at which hæmorrhage occurs; sometimes it has occurred before that. In placenta prævia the advance of the placenta through the os internum begins at about seven months, and I conclude that the os internum usually begins to open up at the seventh month.

### Varieties.

In placenta prævia the placenta is usually more on one side than on the other. There is *central* placenta prævia, in which the placenta overlaps the os uteri all round; there is *lateral* placenta prævia, in which only one edge of it is over the os uteri; and there is the *marginal* form, in which the edge of the placenta overlaps the lower zone, and is so low down that the patient cannot be delivered without the placenta being detached. When the placenta is prævia, the usual way in which labour progresses is that the side of the placenta which is least extensively attached becomes stripped off, and so a central placenta prævia becomes converted into a partial placenta prævia. If the placenta prævia is quite central, and the uterus is acting very powerfully, it drives the placenta on, and may separate it altogether, so that it is born before the child. This is a rare event, because for its occurrence there must be strong pains; and strong pains favour safe delivery in placenta prævia.

Sir James Simpson proposed to treat placenta prævia by separating the placenta all round. It is impossible to do this, because the placenta measures usually about 7 inches across, and with your finger in the vagina you cannot reach far enough. The placenta being firmly attached around the os uteri, there is difficulty in stripping it off; and therefore the advancing part of the child does not press on the os uteri and produce a reflex effect, as in natural labour. That is why delivery in placenta prævia is slow; from close adhesion of the lower pole of the ovum. The part to which the placenta is attached is very vascular, and for that reason if it is injured free hæmorrhage is apt to occur. As the ovum advances utero-placental vessels are torn, there is bleeding, and in time those vessels become sealed by thrombosis. At a later date further advance takes place, more vessels are torn through, and are gradually sealed by thrombosis. If the hæmorrhage takes place early—sometimes it is as early as the sixth month—the uterus is not so vascular as it is later, and the bleeding is not so severe. When a patient with placenta prævia has her first hæmorrhage at or near term, the condition is very obvious and the hæmorrhage is copious. The physiology of placenta prævia is that the membranes cannot advance into the os uteri until part of the placenta has been separated.

### Hæmorrhage.

Hæmorrhage is the great and immediate danger to the mother in placenta prævia. There is also danger to the child, because it is prematurely born. Separation of the placenta over a large area means that the child is deprived of its proper supply of oxygen. The fact of the child being premature means that it is more easily killed than a full-term child. Often such a child is in an abnormal position and is delivered with the breech first. If slowly, the cord is pressed on, and that contributes to the death of the child. In former days, before the advent of Listerism, placenta prævia was often fatal from sepsis, especially in lying-in hospitals. Accoucheurs who practised among well-to-do patients found the mortality not so high as it was in those hospitals. The liability of the cervix to injury made the patient much exposed to septic poisoning. An indirect danger to which placenta prævia exposes the patient is that of insanity, arising from three conditions: If the patient has been septically poisoned—she is more liable to become insane than if she had had a healthy lying-in. Secondly, she is anæmic, and anæmia is a condition which predisposes the patient to mental failure. Thirdly, in addition to being feverish and anæmic she is disappointed and depressed at the loss of her baby. These things combined favour insanity. In these several ways placenta prævia is a dangerous condition.

### Treatment.

Now I come to speak of the treatment. First of all, I would say I still assume the proposition which, it appears, is not accepted on the other side of St. George's Channel, that the life of the mother is very much more valuable than that of the child. The mother has a definite place and a definite value to many people; but the child has no value. The child is a possibility, a potentiality, but nothing beyond. The mother's life should always be put before the child's. If the placenta prævia is over the os uteri, there is no possibility of the child being born without hæmorrhage, and you can never tell how great the next hæmorrhage may be. One immediately fatal hæmorrhage is not common in placenta prævia, but such an event does occur. And remember that the next bleeding may occur at a time and place where the patient cannot get proper attention. Therefore, the first principle of treatment which I submit to you is, that when placenta prævia has been diagnosed, induce labour at once. There is no room for any such thing as expectant treatment of placenta prævia. If the patient has a slight hæmorrhage you may not be able to make the diagnosis. Sometimes the placenta can be detected by abdominal palpation. It has been done, but it is so dependent on favourable conditions that you cannot always feel sure you are right. But when you have felt the placenta and know the case to be one of placenta prævia, induce labour. The mortality of placenta prævia was lowered when antiseptic treatment was applied to midwifery. The proper treatment of placenta prævia was taught clearly and emphatically by Braxton Hicks; and when, on the Continent, they applied Hicks's methods, the mortality at once fell. At one time it was 20 per cent., and it fell to less than 5 per cent. Braxton Hicks instructed the profession as to the treatment of placenta prævia, but his treatment was not new. Madame La Chapelle taught it a century ago. At that time antiseptics were not known. She recommended turning, bringing down the foot, and delivering gently. The half breech is a soft pad, which presses on the bleeding vessels and so stops the hæmorrhage. If the child is extracted slowly the mother is not injured. If you have no instruments, that still remains the best treatment. As soon as you have made the diagnosis, bring down the foot, watch the case, and do not hurry. The pressure of the breech in the os uteri will check hæmorrhage and stimulate the uterus to action. The objection to this treatment is that it involves a fœtal mortality of about 90 per cent. The mortality is sure to be high whatever you do in placenta prævia, because the child is premature and small, and may be asphyxiated from want of oxygen, which should be supplied to it through the placenta.

Champetier's bag is a good way of bringing about delivery. When that bag has done its work you can deliver the child easily and quickly without its cord being pressed on, and therefore it has a fair prospect of living. An objection brought against that bag is that it may displace the head. That is correct, but it does not matter, because you can immediately turn and deliver. Robert Barnes, in his book on obstetric operations, gives directions. He says: Separate the placenta all round, and then "often the hæmorrhage ceases." I am afraid that sentence of Robert Barnes's has caused a good deal of misunderstanding in the minds of practitioners; and that here and there a woman has been sacrificed to it, because the reader has got it into his mind that if he separates the placenta the hæmorrhage will cease. Barnes is right in saying "hæmorrhage *often* ceases," but *not always*. When the placenta is attached round the os uteri it must be separated before the child can descend. If you separate all round, you make the advance of the ovum easier. Barnes was not the first to recommend separation of the placenta, but I think he was the first to separate the placenta all round. Still, I do not think he clearly saw, at least he did not state in his book, the advantage of doing it.

### Septic Conditions.

Separate the placenta all round as far as you can reach. When the centre of the placenta is not exactly over the os uteri, your finger will at one side pass the edge of the placenta and come upon the membranes. If you have no bag with you, perforate the membranes at that point and bring down a foot. If you have Champetier's bag pass it up. The bag raises the tension in the uterus, and provokes uterine action. With the bag you are able to prevent further hæmorrhage. Then it is important that the extraction should be slow, because if you extract the child quickly that means that the cervix uteri, which is very vascular, is torn more than it need be; and that tear means hæmorrhage, because the placental site is very vascular. The danger of tearing is not imaginary. I have had to repair a vesico-vaginal fistula caused by violent delivery in a case of placenta prævia.

I have spoken about the danger of septic infection. To conduct labour aseptically, in the literal sense of the word, is an impossibility. You cannot prevent germs being present; they are everywhere. But you can reduce the dose of germs to one with which the organism can deal. At many lying-in hospitals now placenta prævia is treated with a mortality below 5 per cent. They do not get septic afterwards.

### Operative Interference.

The latest proposal for the treatment of placenta prævia is Cæsarean section. When delivery is effected by the vagina, the mortality in placenta prævia is something under 5 per cent. In 1904 the mortality of Cæsarean section in the best hospitals in Europe was 8 per cent. Of all the conditions in which Cæsarean section might be thought suitable, I think the least suitable is placenta prævia. What are the advantages of Cæsarean section in a labour which is expected to be difficult? The great advantage of it, as compared with other methods of delivery, is that you put aside all mechanical difficulty. When the head is above the brim and you try delivery by forceps it may be too big, and the child will not be born alive. But these things are put away at once when you do Cæsarean section. In placenta prævia there is no mechanical difficulty. The child is premature and small, can be got through the pelvis with the greatest ease, and therefore Cæsarean section is an unnecessary procedure from the mechanical point of view.

There are other points with regard to Cæsarean section. I think the operation will in future have a far less mortality than 8 per cent. I cannot tell you why it has such a high mortality. Cæsarean section is the one operation in which all the parts are healthy. It is like an ordinary operation done on a healthy man, who is not damaged by shock or anything else. In Cæsarean section the parts are healthy, and every part is where it ought to be, and every step can be foreseen and provided for. It is only the amount of accident due to human error which causes there to be any mortality at all. But, no doubt, as proficiency in operating and care in the matter of antisepsis increase, the mortality from Cæsarean section will become very low. If there is a condition of danger from Cæsarean section it is where there is abnormality in the vascular supply of the parts, and that is the condition in placenta prævia. So I do not think there is an advantage in treating placenta prævia by Cæsarean section.

### Vaginal Plugging.

There is another method of treatment which I should have mentioned, and that has been told us from the other side of the water, as if it were something very novel. That is plugging the vagina. They tell us there that in accidental hæmorrhage it is the thing. I have no belief whatever in plugging the vagina in placenta prævia. It is a clumsy thing, and one can only say that it does some little good as a stimulant of the uterus. If you plug the vagina tightly, it causes the patient great pain, and you may be sure you will not get the opportunity of doing it again on that patient. You perhaps fill the vagina with gauze, as much as you can; but if you examine the patient in an hour or two you will find the gauze has been converted into a smooth ball, which slips about in the vagina, and blood easily flows past it. Plugging is nothing new. It has been debated about for 150 years. Madame La Chapelle told her pupils that they might plug the vagina, but that it was not necessary to tie together the bits of charpie, because there was no difficulty in getting them out again, for after a time the vagina stretches; it is not of such cartilaginous consistence as the uterus; and the finger can get past each plug of charpie and hook it down. Madame La Chapelle was at one with Braxton Hicks, who condemned the useless pain which is given the patient by plugging.

The way of stopping hæmorrhage in placenta prævia is to dilate the os by means of gentle continuous pressure upon the lower segment of the uterus. The best way to secure that pressure is with the Champetier water-bag, or some other kind. If you have not this, you can get a toy indiarubber bag for a penny at a shop. If you have not a bag, bring down the breech of the child so that it will press on the bleeding area and stop the hæmorrhage. Induce labour as soon as placenta prævia has been diagnosed. Having brought down the foot, pull on it gently, only enough to stop the hæmorrhage. The presence of the bag will stimulate the uterus to contract and stop the bleeding. Let the labour go on as naturally as possible, using no violence to extract the child.

### Conclusions.

You cannot do anything until you have found out that the case is one of placenta prævia, and this means that so much dilatation must have taken place as will admit your finger. Placenta prævia is not common in first pregnancies. We know nothing as to the cause of placenta prævia. I think from *a priori* reasoning that the cause of placenta prævia must be some condition of the endometrium. What that condition is, however, no one knows. We do not know how to prevent it, nor how to predict when a patient is three months pregnant that the placenta will be prævia. Prediction and prevention are the great objects which we ought to have in view. In most cases by the time there has been hæmorrhage enough to alarm the friends the os uteri is big enough to admit the finger with which you can feel the placenta. But you cannot bring down the foot with one finger; you must grasp the foot either with one finger and an instrument, or with two fingers. The best way is to dilate the os uteri at once with one finger until you can get two in; separate the placenta, seize the foot, and bring it down. Forceps is seldom of use in placenta prævia. Now and then, if there is a marginal or partial placenta prævia in which the os uteri is dilated to four-fifths of its size, and there has been hæmorrhage going on which has weakened the patient, it may be a good thing to assist the uterus by applying forceps and pulling. But such cases are not frequent, and in such a case you had better not wait for the os uteri to get dilated, but bring down the foot as soon as there is no doubt about the presence of placenta prævia.

---

Next week's Clinic will be on **Tobacco Amblyopia** by Mr. Percy Dunn, F.R.C.S.

